# Mannose receptor may be involved in small ruminant lentivirus pathogenesis

**DOI:** 10.1186/1297-9716-43-43

**Published:** 2012-05-16

**Authors:** Helena Crespo, Paula Jauregui, Idoia Glaria, Leticia Sanjosé, Laura Polledo, Juan F García-Marín, Lluís Luján, Damián de Andrés, Beatriz Amorena, Ramsés Reina

**Affiliations:** 1 Institute of Agrobiotechnology, CSIC-UPNA-Government of Navarra, Ctra Mutilva, Mutilva Baja, 31192, Spain; 2 Veterinary Faculty, University of León, León, 24071, Spain; 3 Department of Animal Pathology, University of Zaragoza, Miguel Servet 177, Zaragoza, 50013, Spain; 4 Present address: Chair of Hunting and Fishing Resources, University of Córdoba, Ctra Nacional IV-a Km 396, Córdoba, 14071, Spain

## Abstract

Thirty-one sheep naturally infected with small ruminant lentiviruses (SRLV) of known genotype (A or B), and clinically affected with neurological disease, pneumonia or arthritis were used to analyse mannose receptor (MR) expression (transcript levels) and proviral load in virus target tissues (lung, mammary gland, CNS and carpal joints). Control sheep were SRLV-seropositive asymptomatic (*n* = 3), seronegative (*n* = 3) or with chronic listeriosis, pseudotuberculosis or parasitic cysts (*n* = 1 in each case). MR expression and proviral load increased with the severity of lesions in most analyzed organs of the SRLV infected sheep and was detected in the affected tissue involved in the corresponding clinical disease (CNS, lung and carpal joint in neurological disease, pneumonia and arthritis animal groups, respectively). The increased MR expression appeared to be SRLV specific and may have a role in lentiviral pathogenesis.

## Introduction, methods and results

Pneumonia and mastitis have been the most common small ruminant lentivirus (SRLV)-triggered clinical forms found in Spanish sheep flocks [1], but two disease outbreaks have been described recently, either targeted to the CNS (visna), involving meningoencephalitis [2] and myelitis [3], or causing arthritis, specifically carpal joint synovitis, together with interstitial mastitis and/or pneumonia [4,5]. Histopathologically, the different SRLV disease forms involve lesions with infiltration of macrophages and lymphocytes, non-suppurative inflammation and fibrosis [6]. Virus levels have been positively correlated with lesion severity in terms of proviral load in blood [7], proviral and viral load in tissues [8] or proviral load in lungs [9]. In this study, knowing the role of the mannose receptor (MR) as a cellular receptor for SRLV in vitro [10], we aimed to investigate in SRLV natural infections if MR expression was increased in affected target tissues from animals with the different clinical forms of the disease and if this putative increase was related to the presence of infected cells (increased proviral load) and lesions in these tissues, in order to gain knowledge on factors involved in the development of SRLV-induced clinical infection and pathogenesis.

Thirty-one sheep naturally SRLV-infected according to Elitest (Hyphen Biomed, France), which were also clinically affected (Table 1), were humanely euthanatized in compliance with the current European and national (RD 1201/2005) regulations, with the approval of the “Comité de Ética y Experimentación Animal” of the Universities of León and Zaragoza and authorization of the Castilla y León and Aragón Governments for pathology studies. Sheep were distributed into three groups for comparative purposes according to clinical manifestations: a) encephalitis (with non-purulent encephalitis, myelitis and/or choroiditis/meningitis); b) pneumonia (affected by interstitial pneumonia and follicular hyperplasia in lungs); and c) arthritis (with arthritis in carpal joints, in the presence/absence of pneumonia and/or mastitis). Three seropositive asymptomatic and three seronegative animals, included as controls, did not show any lesion compatible with SRLV infection. Tissue samples from lung, mediastinic lymph node, central nervous system (brain cortex, diencephalon, corpus callosum, hippocampus, midbrain, cerebellar cortex, pons and cerebellar peduncles, medulla oblongata, and cervical, thoracic and lumbar spinal cord), carpal joint and mammary gland were collected post-mortem and stored at −80°C in RNAlater (Qiagen) or fixed in 10% buffered formalin and zinc fixative salts (0.5% zinc chloride, 0.5% zinc acetate in 0.1 M Tris base buffer containing 0.05% calcium acetate, pH = 7.4) for subsequent staining with hematoxylin-eosin and examination under light microscopy by two different pathologists.

**Table 1:** Histopathological post-mortem observations in SRLV target tissues among groups of animals clinically affected with encephalitis, pneumonia or arthritis

		**Tissue**						
**Clinical disease**	**Animal no.**	**CNS**			**Lung**		**Carpal joints**	**Mammary glands**
		**E**	**C**	**ME**	**IP**	**FH**	**A**	**IM**
Encephalitis	1	+	+	+++	-	-	-	+
(*n* = 15)	2	+	-	+++	-	-	-	Male
	3	+	++	+++	-	+	-	+
	4	+++	+	-	-	-	-	-
	5	+++	+	+++	-	-	-	-
	6	++	+++	+++	-	-	-	-
	7	+	+++	-	-	-	-	+
	8	+	++	-	-	-	-	-
	9	+	++	-	-	-	-	-
	10	+++	+	++	-	+	-	+
	11	+++	+	-	-	+	-	-
	12	+++	+	-	-	-	-	+
	13	+	+++	++	-	+	-	-
	14	+++	+	+++	-	-	-	++
	15	+++	+	+++	-	-	-	++
Pneumonia	16	-	-	-	+++	+++	-	+
(*n* = 8)	17	-	-	-	++	++	-	-
	18	-	-	-	+	+	-	-
	19	-	-	-	++	++	-	+
	20	-	-	-	+	+++	-	-
	21	-	+	-	++	+++	-	-
	22	-	-	-	++	+++	-	+
	23	-	-	-	+	NA	-	-
Arthritis	24	-	-	-	+++	++	+++	-
(*n* = 8)	25	-	-	-	+	-	+	-
	26	-	-	-	+	-	+	++
	27	-	-	-	++	++	++	++
	28	NA	NA	NA	NA	NA	NA	NA
	29	-	-	-	+	+	+	++
	30	-	-	-	+	+++	+	-
	31	NA	NA	NA	NA	NA	NA	NA

Signs of lesion were recorded as encephalitis (E), choroiditis (C), myelitis (ME), interstitial pneumonia (IP), follicular hyperplasia (FH), arthritis (A) and interstitial mastitis (IM). The severity of lesions was scored as (−) absence of lesion, (+) mild, (++) moderate, (+++) severe. NA: Not available.

Scoring of histological VMV-related lesions in target organs were based on previous works [11]. The parameters evaluated in lungs were the presence of interstitial pneumonia (IP) as: “-”absent; “+” mild and “++”severe, and follicular hyperplasia (FH) as: “-”absent; “+” < 2 follicles; “++” 3–6 follicles; “+++” > 6 follicles per 40x·field. In mammary glands, the interstitial mastitis (IM) was evaluated as in lungs. In carpal joints, the severity of the arthritis (A) was evaluated as “-“absent; “+” light; “++” mild and “+++” severe. Lesions of the nervous system were classified into the previously described categories [2]: “-”absent; “+” mild, consisting of multifocal small areas of gliosis; “++” moderate, consisting of multifocal to coalescent zones of non-suppurative inflammation, composed of perivascular cuffs of lymphocytes and macrophages with limited areas of demyelination; and “+++” severe, characterized by extensive areas of diffuse mononuclear inflammation, with numerous macrophages and malacia.

**Figure 1:**
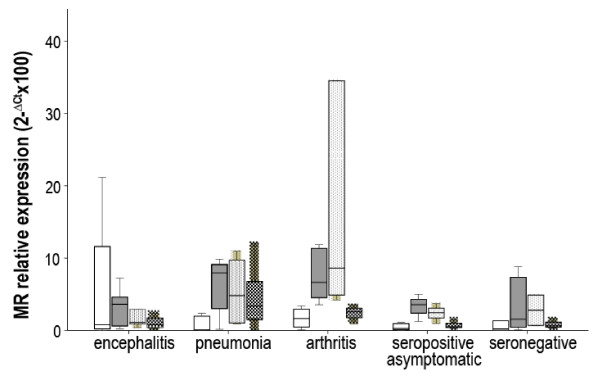
**MR relative expression in SRLV target tissues from animal groups affected with neurological disease (indicated as encephalitis), pulmonary disease (pneumonia) or arthritis. Seropositive asymptomatic and seronegative animals were included as controls.** Target tissues were CNS structures (open bars), respiratory tract (grey bars), carpal joints (dotted bars) and/or mammary gland (squared bars). Data are expressed are expressed as 2- ΔCt ☒ 100 Â± SE.

Tissue samples (10 mg pieces) were disrupted using the Mikro-Dismembrator U (Sartorius) in AL buffer (Qiagen) or RLT buffer plus ß-mercaptoethanol for DNA and RNA extraction, respectively. DNA was extracted with Qiamp DNA Blood Mini kit (Qiagen) and RNA with RNeasy Mini Kit (Qiagen). Then RNA was treated with DNAseI (Sigma) and retrotranscribed to cDNA (RT-PCR) with SuperScript II (Invitrogen), using oligo-dT as primers to determine MR expression (transcripts) in tissues by real time PCR as described previously [10], using ß-actine as housekeeping gene for each cDNA sample. MR expression values (2^-ΔCt^ × 100) and proviral load (viral DNA copies/100 ng of DNA; see below) were compared by non-parametric Wilcoxon test for related samples and comparisons between groups were made (non-related samples) by Mann-Whitney’s test. Within each diseased animal group, real time PCR results revealed a significantly increased MR expression in SRLV affected tissues (Figure 1): CNS (*p* = 0.0145), lung (*p* = 0.024) and carpal joint (*p* = 0.001) in the encephalitis, pneumonia and arthritis groups, respectively. In line with this, inter-group analysis indicated that, compared to other animal groups, CNS samples had the highest MR expression in the encephalitis group (*p* = 0.035); and carpal joints had the highest MR expression in the arthritic group (*p* = 0.013). Although lung and mediastinic lymph node appeared to have the highest MR expression in the pneumonia group compared to others, differences were only statistically significant when this group was compared with the encephalitis group (*p* = 0.020). The presence of mastitis was not evident macroscopically, but in the three groups (Table 1) there were animals presenting mild or moderate (never severe) SRLV-compatible microscopic lesions in the mammary gland. MR levels were higher in animals presenting lesions in this organ (moderate alone or combined with mild) compared to animals not presenting lesions (*p* = 0.034); similarly in lungs, MR expression was increased in animals with severe lung lesions in comparison with those without lesions (*p* = 0.021).

**Figure 2:**
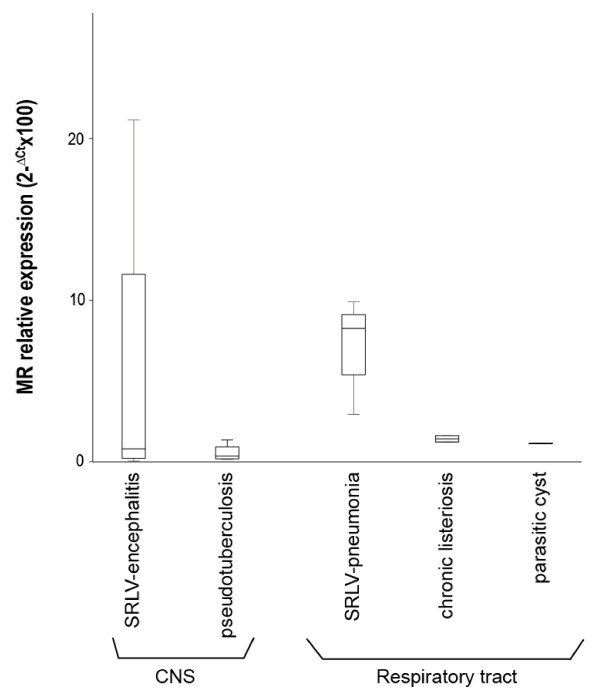
**MR expression in CNS structures of animals with neurological disease caused by SRLV (*n*** **= 15) or by chronic listeriosis (*n*** **= 1) and in respiratory tract (lung and mediastinic lymph node, ML) of animals affected with SRLV pneumonia (*n*** **= 8), pseudotuberculosis (*n*** **= 1), and lung parasitic cysts (*n*** **= 1).** Data are expressed as 2^- ΔCt^ × 100 ± SE.

**Figure 3:**
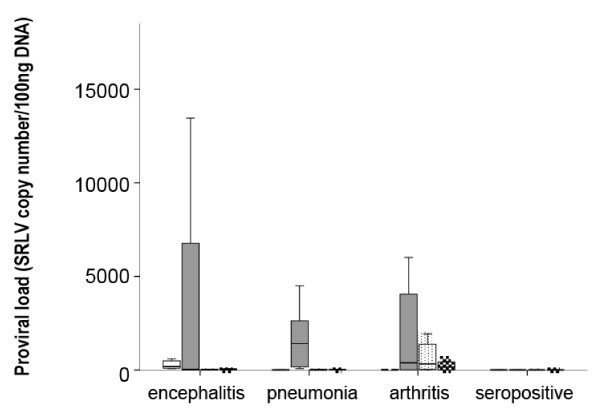
**Proviral load (SRLV copy number/100 ng of DNA) in target tissues (CNS structures (white bars), respiratory tract (grey bars), carpal joint (dotted bars), and mammary gland (squared bars) among the clinically affected groups.** Proviral load was quantified by real-time PCR amplifying the LTR region.

**Figure 4:**
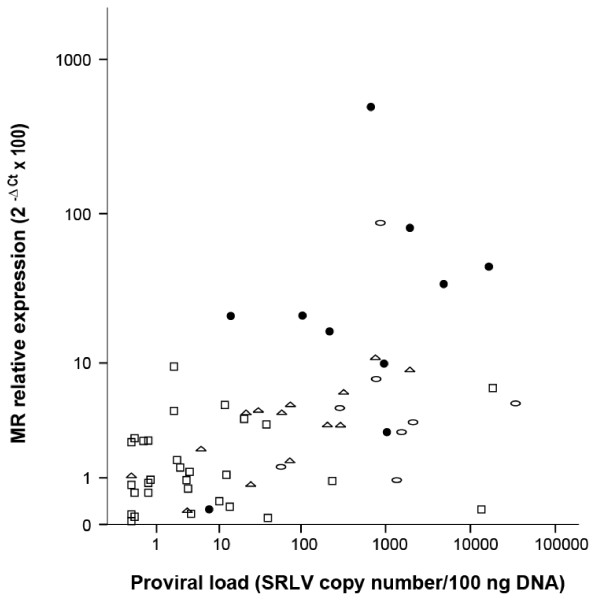
Relationship between MR relative expression (2-ΔCt ☒ 100) and proviral load (SRLV copy number/100 ng) in target tissues (lung, encephalon, carpal joint and mammary gland) according to the severity of lesions (no lesion (open squares), mild (open triangles), moderate (open circles), or severe (solid circles)) detected in each tissue by histopathological analysis.

Induction of MR expression in affected target tissues appeared to be mediated by SRLV infection, since MR expression in the SRLV-uninfected animals of the control group clinically affected with pseudotuberculosis, pulmonary parasitic cysts (both pulmonary pathologies) or chronic listeriosis (CNS pathology), was low in lung and CNS, respectively, compared with the SRLV-clinically affected animals (Figure 2). Accordingly, proviral load was measured in affected tissues to further investigate a possible link between SRLV infection and MR expression. Specifically, proviral load was assessed by real time PCR of genomic DNA as described previously [12], using SYBR Premix Ex Taq (Takara) and LTR-region specific primers designed for encephalitis A [13], pneumonia A [14] and arthritis B2 (this work; Fw: TGCTGCTTGCACTTCRGAGTT; Rv: GGCAGTAAGGCAATCACTCCTT) genotypes to obtain amplicons of 471, 233 and 98 nt, respectively. Serially diluted plasmids (pGEM-T Easy, Promega) containing the LTR region were employed to generate the standard curve (Ct vs. copy number) and determine sample copy number values. Plasmid copy number ranged from 1 to 10^6^. Results, expressed as provirus copy number/100 ng of DNA, indicated that in all the clinically affected groups, the highest proviral load was detected in lung (Figure 3). When studying other tissues, the results resembled those on MR expression in that within each particular animal group, the highest number of copies (proviral load in this case) corresponded to tissues with main lesions, particularly CNS structures in the encephalitis group (*p* < 0.05), respiratory tract in the pneumonia group (*p* < 0.05), and carpal joints for the arthritic group (*p* < 0.02). Also, inter-group comparisons revealed an increased (*p* < 0.005) proviral load in CNS structures of encephalitis-affected animals compared to other groups, as observed in MR expression. The proviral load in mammary glands or lungs did not differ between the three clinically affected groups described in Table 1, but when animals of these groups were jointly reclassified attending to lesion degree (null, mild, moderate or severe), as it was done when assessing MR level differences, not only the presence or absence of lesions but also the degree of lesion was significantly associated with proviral load, as observed in MR expression. For example, mammary gland proviral load was increased either in animals with moderate lesions in this organ compared to those with mild lesions (*p* = 0.016) or in animals with lesions in this organ compared to those without lesions (*p* = 0.003). In line with this, lung proviral load was increased in lung with severe (*p* = 0.002) or with any degree of lesion (*p* = 0.003) compared to lung without lesions. Since different levels of lesion severity were recorded in target organs, all MR expression and proviral load values were plotted according to these levels of severity (Table 1). In the case of lung or CNS, where more than one sign were evaluated per organ, only one, FH and E respectively, were considered as representative of the overall lesion degree in that specific organ (Figure 4). Severe lesions were mainly (7 out of 9 cases) accompanied by increased MR expression and increased proviral loads. There were organs presenting moderate lesions which also showed both, increased MR and proviral load values, strongly suggesting intermediate steps in the process. Although there were exceptions, two organs with a high proviral load, low-to-very low MR expression and no lesions, and one organ with low MR expression and viral load but severe lesions (Figure 4).

## Discussion

MR is one of the putative SRLV receptors and, although MR transcripts have been found in vitro in sheep monocytes/macrophages and synovial membrane fibroblasts [10], the levels of MR transcripts in SRLV target tissues from natural clinical infections have not been assessed so far and it is unknown if MR could play a role in SRLV pathogenesis. This work demonstrates that MR expression (transcript levels) and proviral load are increased in tissues affected by clinical A or B SRLV infections presenting different disease forms (CNS in the neurological, lung in the pulmonary and carpal joint in the arthritic form), and that the increase in both of them is related to the severity of lesions, strongly suggesting a role of MR expression in SRLV pathogenesis, independently of the disease form presented by the animal. Increased proviral load in affected organs was expected according to previous SRLV results [7-9]. In addition, the increased MR expression observed in heavily infected body sites would be expected, since the virus may enter the cell via MR [10,15] and cells like macrophages with enhanced MR expression may not favour Th1 responses (as observed in mice; [16]), which would be necessary for development and differentiation of cytotoxic cells against virus infected targets. Thus, MR-expressing macrophages may enhance clinical disease development.

The scattered distribution of mammary gland lesions into the three clinically affected study groups together with the absence of severe mastitis lesions, may explain the lack of significant differences in MR expression and viral DNA load in mammary gland between the three clinically affected study groups. This is in line with the existence of significant differences in MR expression and proviral load demonstrated between animal groups when animals were instead classified according to lesion severity. Comparative analysis of lesions distribution according to lesion degree, levels of MR and proviral load further indicated a positive association between these three parameters. Interestingly, the organ found with no lesion but high proviral load and very low MR expression was the lung from animal 14, which presented severe lesions in other organs (Table 1), indicating that persistent infection was already established and clinical manifestations would appear progressively in this target organ. Similarly, the absence of lesion found in an organ (lung) with high MR/proviral load corresponded to animal number 12 that showed another type of lesion (IP), suggesting an incipient stage in the development of clinical signs in the lung of this animal. Another interesting organ was that which presented severe lesions but low MR expression and proviral load. Analysis of all the tissues under study from the corresponding animal (number 5) indicated always low MR and proviral load, suggesting a possible role for the host’s genetic background. As revealed by proviral load values, infected cells appeared to be more abundant in the lungs of all groups, which was expected since lung-associated cells may represent one of the main infection routes [17] favouring viral replication in alveolar macrophages [18].

The identification of MR as a main cell entry pathway involved in pathogenesis opens possibilities in entry-blocking treatment against SRLV and new insights into SRLV control beyond diagnosis and immunization.

## Abbreviations

CNS: Central nervous system; MR: mannose receptor; SRLV: small ruminant lentiviruses; ML: Mediastinic lymph node.

## Competing interests

The authors declare that they have no competing interests.

## Author’s contributions

HC carried out the PCR and RT-PCR studies and drafted the manuscript. PJ, IG, and LS participated in tissue processing, RNA preparation and cDNA obtention. LL, LP and JFGM carried out the histopathological evaluation, clinical classification and obtained tissue samples. DA and BA searched for funding resources and were involved in the focusing, writing and discussion of the manuscript. RR conceived and designed this study, being involved in work supervision and writing of the manuscript. All authors read and approved the final manuscript.

## References

[B1] Lujan L Garcia Marin JF Fernandez de Luco D Vargas A Badiola JJ Pathological changes in the lungs and mammary glands of sheep and their relationship with maedi-visna infection Vet Rec 1991 129 51 54 10.1136/vr.129.3.51 1656581

[B2] Benavides J Gomez N Gelmetti D Ferreras MC Garcia-Pariente C Fuertes M Garcia-Marin JF Perez V Diagnosis of the nervous form of Maedi-Visna infection with a high frequency in sheep in Castilla y Leon, Spain Vet Rec 2006 158 230 235 10.1136/vr.158.7.230 16489160

[B3] Benavides J Fuertes M Garcia-Pariente C Ferreras MC Garcia Marin JF Perez V Natural cases of visna in sheep with myelitis as the sole lesion in the central nervous system J Comp Pathol 2006 134 219 230 10.1016/j.jcpa.2005.11.003 16615937

[B4] Biescas E Contribución al estudio serológico, lesional, diagnóstico y preventivo del Maedi Visna ovino PhD thesis 2006 University of Zaragoza

[B5] Glaria I Reina R Crespo H de Andrés X Ramirez H Biescas E Perez MM Badiola J Lujan L Amorena B de Andrés D Phylogenetic analysis of SRLV sequences from an arthritic sheep outbreak demonstrates the introduction of CAEV-like viruses among Spanish sheep Vet Microbiol 2009 138 156 162 10.1016/j.vetmic.2009.03.002 19339126

[B6] Pépin M Vitu C Russo P Mornex JF Peterhans E Maedi-visna virus infection in sheep: a review Vet Res 1998 29 341 367 9689746

[B7] Herrmann-Hoesing LM Noh SM White SN Snekvik KR Truscott T Knowles DP Peripheral ovine progressive pneumonia provirus levels correlate with and predict histological tissue lesion severity in naturally infected sheep Clin Vaccine Immunol 2009 16 551 557 10.1128/CVI.00459-08 19261772 PMC2668279

[B8] Ravazzolo AP Nenci C Vogt HR Waldvogel A Obexer-Ruff G Peterhans E Bertoni G Viral load, organ distribution, histopathological lesions, and cytokine mRNA expression in goats infected with a molecular clone of the caprine arthritis encephalitis virus Virology 2006 350 116 127 10.1016/j.virol.2006.02.014 16537085

[B9] Zhang Z Watt NJ Hopkins J Harkiss G Woodall CJ Quantitative analysis of maedi-visna virus DNA load in peripheral blood monocytes and alveolar macrophages J Virol Methods 2000 86 13 20 10.1016/S0166-0934(99)00169-X 10713371

[B10] Crespo H Reina R Glaria I Ramirez H de Andrés X Jauregui P Lujan L Martinez-Pomares L Amorena B de Andrés DF Identification of the ovine mannose receptor and its possible role in Visna/Maedi virus infection Vet Res 2011 42 28 10.1186/1297-9716-42-28 21314911 PMC3041668

[B11] Salazar E Monleon E Bolea R Acin C Perez M Alvarez N Leginagoikoa I Juste R Minguijon E Reina R Glaria I Berriatua E de Andrés D Badiola JJ Amorena B Lujan L Detection of PrPSc in lung and mammary gland is favored by the presence of Visna/maedi virus lesions in naturally coinfected sheep Vet Res 2010 41 58 10.1051/vetres/2010030 20423698 PMC2881419

[B12] Reina R Glaria I Benavides J de Andrés X Crespo H Solano C Perez V Lujan L Perez MM de la Lastra JM Perez Rosati S Blacklaws B Harkiss G de Andrés D Amorena B Association of CD80 and CD86 expression levels with disease status of Visna/Maedi virus infected sheep Viral Immunol 2007 20 609 622 10.1089/vim.2007.0071 18158734

[B13] Glaria I Reina R Ramirez H de Andrés X Crespo H Jauregui P Salazar E Lujan L Perez MM Benavides J Perez V Polledo L Garcia-Marin JF Riezu JI Borras F Amorena B de Andrés D Visna/Maedi virus genetic characterization and serological diagnosis of infection in sheep from a neurological outbreak Vet Microbiol 2012 155 137 146 10.1016/j.vetmic.2011.08.027 21940116

[B14] Zanoni R Phylogenetic analysis of small ruminant lentiviruses J Gen Virol 1998 79 1951 1961 9714243 10.1099/0022-1317-79-8-1951

[B15] Ezekowitz RA Williams DJ Koziel H Armstrong MY Warner A Richards FF Rose RM Uptake of Pneumocystis carinii mediated by the macrophage mannose receptor Nature 1991 351 155 158 10.1038/351155a0 1903183

[B16] Mantovani A Sica A Sozzani S Allavena P Vecchi A Locati M The chemokine system in diverse forms of macrophage activation and polarization Trends Immunol 2004 25 677 686 10.1016/j.it.2004.09.015 15530839

[B17] Ramirez H Study of compartimentalization in the visna clinical form of small ruminant lentivirus infection in sheep BMC Vet Res 2012 8 8 10.1186/1746-6148-8-8 22281181 PMC3328241

[B18] Lairmore MD Akita GY Russell HI DeMartini JC Replication and cytopathic effects of ovine lentivirus strains in alveolar macrophages correlate with in vivo pathogenicity J Virol 1987 61 4038 4042 2824834 10.1128/jvi.61.12.4038-4042.1987 PMC256029

